# Exploring the ‘citizen organization’: an evaluation of a regional Australian community-based palliative care service model

**DOI:** 10.1177/26323524241260427

**Published:** 2024-06-24

**Authors:** John Rosenberg, Trudi Flynn, Katharina Merollini, Josie Linn, Doreen Nabukalu, Cindy Davis

**Affiliations:** University of the Sunshine Coast, Tallon Street, Caboolture, QLD 4510, Australia; School of Law and Society, University of the Sunshine Coast, Sippy Downs, QLD, Australia; School of Health, University of the Sunshine Coast, Sippy Downs, QLD, Australia; School of Health, University of the Sunshine Coast, Sippy Downs, QLD, Australia; School of Health, University of the Sunshine Coast, Petrie, QLD, Australia; School of Law and Society, University of the Sunshine Coast, Sippy Downs, QLD, Australia

**Keywords:** citizen health, community care, community engagement, hospice, model of care, palliative care

## Abstract

**Background:**

Little Haven is a rural, community-based specialist palliative care service in Gympie, Australia. Its goals are to provide highest quality of care, support and education for those experiencing or anticipating serious illness and loss. Families and communities work alongside clinical services, with community engagement influencing compassionate care and support of dying people, their families and communities. Public Health Palliative Care promotes community engagement by community-based palliative care services and is grounded in equal partnerships between civic life, community members, patients and carers, and service providers. This takes many forms, including what we have termed the ‘citizen organization’.

**Objectives:**

This paper reports on an evaluation of Little Haven’s model of care and explores the organization’s place as a ‘citizen’ of the community it services.

**Design:**

A co-designed evaluation approach utilizing mixed-method design is used.

**Methods:**

Multiple data sources obtained a broad perspective of the model of care including primary qualitative data from current patients, current carers, staff, volunteers and organizational stakeholders (interviews and focus groups); and secondary quantitative survey data from bereaved carers. Thematic analysis and descriptive statistics were generated.

**Results:**

This model of care demonstrates common service elements including early access to holistic, patient/family-centred, specialized palliative care at little or no cost to users, with strong community engagement. These elements enable high-quality care for patients and carers who describe the support as ‘over and above’, enabling good quality of life and care at home. Staff and volunteers perceive the built-in flexibility of the model as critical to its outcomes; the interface between the service and the community is similarly stressed as a key service element. Organizational stakeholders observed the model as a product of local activism and accountability to the community.

**Conclusion:**

All participant groups agree the service model enables the delivery of excellent care. The construction of a community palliative care service as a citizen organization emerged as a new concept.

## Plain language summary

‘Citizen organization’: an Australian community-based palliative care service model

Little Haven is a rural, community-based specialist palliative care service in Gympie, Australia. It aims to provide highest quality of care, support and education for those experiencing or anticipating serious illness and loss. Families and communities work alongside clinical services, with community engagement influencing compassionate care and support of dying people, their families and communities. Public Health Palliative Care promotes community engagement by community-based palliative care services in equal partnerships between civic organizations, community members, patients and carers, and service providers. We undertook an evaluation of Little Haven’s model of care by speaking with current patients, current and past carers, staff, volunteers and stakeholders about their experiences of Little Haven. We found that Little Haven’s model contains the essential elements of a palliative care service and provides early access to holistic, patient/family-centred, specialized palliative care at little or no cost to users. They have strong community engagement with a strong background in community activism. We identified that Little Haven’s ‘being in the community’ goes beyond service provision or even sentiment. We observed a symbiotic relationship between the organization and the community it supports in what we have termed the ‘citizen organization’. The distinctive characteristic of the citizen organization is its inseparability from the community in which it dwells.

## Introduction

Palliative care, considered by the World Health Organization (WHO) as a human right, aims at improving the quality of life for patients who are facing life-threatening illness and their families through preventive and treatment measures that relieve pain, reduce suffering and improve psychosocial and spiritual health.^1^ The need for palliative care is significant, with WHO estimating about 26 million people need palliative care each year.^1^ In the Australian context, given approximately 160,000 annual deaths, it is estimated that 75% would benefit from palliative care.^2^ Generally, early provision of palliative care improves end-of-life outcomes, such as alleviating the symptom burden,^3^ reduced pain,^4^ improved satisfaction with care,^5^ reduced use of intensive care units^6^ and reduced length of hospital stay.^7^

Decisions around end-of-life care are complex, and the wishes of patients and their families are crucial in making these critical decisions. Dying at home is a preference for ±80% of people who have never experienced palliative care.^8^ In Australia, 60–70% of people prefer to die at home regardless of age,^8,9^ and ±80% of patients do not change their preference as their disease progresses^10^; this is also evident amongst caregivers and families of palliative care patients.^11^ However, in Australia the settings in which palliative care is provided vary across different regions.^2^ Despite the limited data on palliative care provided outside formal hospital settings, a substantial number of people still receive palliative care within community settings. The Palliative Care Outcomes Collaborative^12^ states that ±52% of *reported* palliative care patients were dwelling in the community, during symptomatic episodes and other phases of care.^13^

The evolution of palliative care services in Australia over many decades has led to enormous variations in models of care:

In Australia, palliative care services have emerged largely as a result of funding initiatives since the 1980s with services operating in outpatient, inpatient and community-based settings, with a combination of public and private providers utilising both specialist and generalist models of care. (Phillips *et al*.,^14^ p. 374)

This has resulted in diverse models which, although they possess common elements (Table 1), vary widely. Little Haven Cooloola Sunshine Coast Palliative Care Association Inc. (Little Haven) is a community-based specialist palliative care and bereavement support service based in Gympie, Queensland, Australia. Its stated goals are ‘to provide the delivery of the highest quality of care, support and education for those experiencing or anticipating serious illness and loss in the Gympie and surrounding region’.^15^ Their model of care recognizes the value of families and communities working alongside clinical services, and the influence community engagement has on compassionate care and support of dying people, their families and communities. Further to providing specialist, interdisciplinary, clinical palliative care, Little Haven promotes early intervention, support of patients during active treatment, and allied health and psychosocial support. These holistic approaches enable optimal independence and planning for anticipated care needs as health declines, and before crises occur, if possible. Little Haven’s early intervention enables a reduction in the avoidable transfer to hospital of home-dwelling patients during their palliative care, which in turn can result in better patient and carer experiences, and efficiencies in the delivery of health care.^16,17^

**Table 1.** Common elements of palliative care service models.

Accessibility of service	Through provision of home visits and after-hours care, 24-h phone support, provision of care on demand and operating day care centres.^18^
Specialist staff	Specialist palliative care teams including palliative care nurses give community palliative care a distinct advantage compared to general home care.^19^
Early intervention	Support for patients early after diagnosis and sometimes during active treatments, including chemotherapy; very effective in improving patients’ outcomes.^20^
Patient- and family-centred approach	Non-professional team members such as friends, family members, volunteer community members and spiritual/faith leaders are encouraged to provide in any way they can. This enables people to spend their last days in the company of those who are important to them.^21^
Holistic care	In community-based models, care goes beyond the physical health of a person and involves spiritual and psychosocial support, bereavement counselling and other services, such as advance care planning and complementary therapies, in addition to medical, nursing and allied health services, with noted benefits to patients.^19^
Community involvement	Services may have teams of community volunteers, such as respite carers and pastoral carers, who are actively involved in the support of patients, fundraising activities and spiritual care.^22^
Free to low-cost services	Most of the organizations are not-for-profit and provide free services, including equipment hire.^23^

Regional and rural communities have palliative care needs distinct from metropolitan communities.^24^ Although some greater community cohesion can be present, under-resourcing and physical distance to health and social care services can impede access to health care; the need for knowledgeable, skilled and engaged communities is critical to effective care and support. Through its community engagement, Little Haven provides care for the partners and families of patients during the care of the dying person and for some time after their death; this bereavement support is a substantial part of Little Haven’s model of care. Little Haven possesses the seven common elements found in most community-based palliative care service models (Table 1). In this evaluation, it was important to understand how models of care impact upon the provision of acceptable, feasible and efficient palliative care. These models must be carefully evaluated for suitability and acceptability to their end users – patients, families and carers – for whom a preordained or assumed approach to care can render it ineffective in achieving their goals of care.^24^

Indeed, receiving palliative care in the community considerably increases the likelihood of death occurring at home where this is concordant with patients’ and carers’ wishes.^25,26^ Patients receiving home-based palliative care generally have reduced demand for hospital-based care and show lessened symptom burden, compared to those receiving general home healthcare; this impact is felt by family caregivers who subsequently experience less complicated grieving.^19,27–30^ Compared to usual care, there is evidence for improved health-related quality of life among patients receiving home-based palliative care.^25,31^ A core tenet of palliative care is the placement of the person and carer at the centre of care, clearly evident in community-based settings where individualized care prevails.

The principle of holistic care originates in the modern hospice movement and is evident in services where person-centred care addresses not just the clinical requirements of a given patient, but the context within which their illness experience occurs. The provision of person-centred, holistic care requires care providers to:

. . .consider people’s relational, creative, spiritual, cultural and social needs, as well as their physical and psychological needs. Strategies to connect on holistic levels can ensure that clinical care aligns with what is meaningful and important to patients and their families. (Stanley and Daddow,^32^ p. 10)

How does Little Haven, as a community-based service, configure its model of care to respond to these preferences and identified needs for care and support? Exploring context with the patient, carer and community enables care to be suitable and acceptable.^24^ With the advent of Public Health Palliative Care, a greater sense of the value of community engagement by community-based palliative care services has emerged, in line with the *Ottawa Charter for Health Promotion* (Box 1).^33^ Public Health Palliative Care is health promoting and requires equal partnerships to operate between the civic life of a community, community members, patients and carers, and service providers.^34^ This takes many forms globally, including what we have termed the ‘citizen organization’ following this evaluation of a regional Australian community-based palliative care service model.

**Box 1.** Ottawa Charter for Health Promotion.^33^

Key action areas: • Build public policies • Create supportive environments • Strengthen community action • Develop personal skills • Reorient health services

### Aim

This study aims to evaluate Little Haven’s model of care and explore the organization’s place as a ‘citizen’ of the wider Gympie rural community.

### Methods

The reporting of this study conforms to the Consolidated Criteria for Reporting Qualitative Research (COREQ^35^; see Supplemental File 1).

### Design

A co-designed evaluation approach utilizing mixed-method design was applied.

### Data collection and analysis

Multiple data sources were used to obtain a broad perspective of Little Haven’s model of care. These are described in Table 2.

**Table 2.** Data sources.

Type	Specific sources
Qualitative in-depth interviews	Current patients (*n* = 6) and carers (*n* = 6) Bereaved family members/carers (*n* = 7) Organizational stakeholders (*n* = 8) including Board Members, Medical Officers (hospital and GPs) and Nursing Director
Qualitative focus groups	Employees (*n* = 10 in three focus groups) including CEO, Clinical Nurse Consultant, Senior Social Worker, RNs and Clinical Nurses Volunteers (*n* = 13 in a single focus group)
Carer Satisfaction Survey	Anonymous mixed-method data from retrospective dataset held by Little Haven (*n* = 116)

GP, General Practitioners.

#### In-depth interviews and focus groups

A range of participant groups were purposively sampled, whose current or past involvement with Little Haven enabled insights through lived experience. Participants included current patients and carers, bereaved carers, staff, volunteers and organizational stakeholders, who were invited to participate in the qualitative components of the research. Purposive recruitment can introduce potential for selection/self-selection bias,^36^ however, rather than seeking clinicians’ vetting, an invitation, the *Research Participant Information Sheet* and *Consent Form* were distributed *via* mail by Little Haven’s administrative service to patients, families and carers. Further, invitations to participate were signposted on the Patients’/Families’ Notice Board in the Little Haven offices, with the *Research Participant Information Sheet* and *Consent Form* in envelopes below the sign. Staff and volunteers were emailed invitations to participate. Organizational stakeholders were identified in the desktop review of Little Haven documentation and were approached individually *via* email and phone by the Chief Investigator.

All interviews and focus groups were audio recorded live or *via* Zoom™ and verbatim transcriptions generated using Otter.ie™ or Zoom™ software and checked for accuracy. Data were coded and categorized with identification of key themes.^36,37^ This paper focuses upon themes that inform the understanding of Little Haven’s model of care and community engagement.

#### Carer Satisfaction Surveys

Carer Satisfaction Surveys are routinely collected by Little Haven from carers/family members 12 weeks after the death of the patient; the CSS instrument is available as a Supplemental File (see Supplemental File 2). Surveys are anonymous and are entered into a database by Little Haven administrative staff to create a longitudinal dataset. Statistical data were transferred into SPSS™ (version 27) for descriptive analysis; narrative analysis of open comments enabled identification of emergent themes informing Little Haven’s model of care and community engagement.

## Results

The multiple sources of data described above provided rich insights into the Little Haven model of care and its place in the community it serves. Results are reported here according to participant group.

### Patients’ and carers’ experiences

Current patients and current and past carers described components of Little Haven’s model of care that supported them to remain at home for most, or all, of their care, and in their bereavement. Initially unaware of Little Haven’s services, ‘I didn’t realise how extensive their support is, and how ongoing and how they really do look after you’ (Bereaved Carer 2), they were equally impressed by the lead role taken in care coordination. Despite the involvement of a large constellation of services, ‘Everyone was on the same page’ (Bereaved Carer 7). Honest and open communication of information by nursing staff alleviated fears and uncertainty for both patients and carers, including in bereavement support.

In turn, this holistic and coordinated approach to care enabled patients and carers to maintain as full a quality of life as possible:

You’ve been told all these things, you’re not really going to make it, mate. You don’t want to keep that in your mind. So you push that out of your mind and try to keep super positive and keep doing all the things that you want to be doing. . .and whether that be things at home to finish them off so that [partner] has still got a lovely home and things are still working for her. . .that’s what is mostly important for me. . . keep busy. . . keep the farm going. And I think keeping all those things, that’s what’s kept my head together as well. (Patient 2)

The provision of holistic, coordinated care included 24-h access to Little Haven clinicians, which was of particular comfort to carers:

I was surprised at how much availability and how supportive and with phone calls and, you know, the 24-hour service. Like if I do have any questions. . .I was surprised that there was something out there like that. So instead of having to go through the hospital system to ask all those questions, . . .everybody that we spoke to nurse-wise [at Little Haven] was able to answer the questions. (Current Carer 1)

Moreover, it enabled the ‘normality’ of home to be preserved even in the presence of concerns requiring clinical intervention. Patients themselves acknowledged that Little Haven’s input went beyond clinical care, and that their quality of life was important to Little Haven too, regardless of the aspect of their lives it sits within; for example, staying on the farm (Patient 2), accessing medication (Patient 3) and staying out of hospital (Patient 4). This support was reported to promote dignity, autonomy and a sense of control in an otherwise uncontrollable circumstance. Continuity of support for bereaved carers was also highly valued, particularly for those whose partner had died and were living alone.

Little Haven’s approach was viewed by many patients and carers to be ‘over and above’ in their advocacy for successful home care: ‘[they’re] going to move heaven and earth to make sure I’m okay’ (Patient 5) and ‘The service provided when needed was way over the top. Dedicated staff who walked the extra mile for their patients and family’ (Survey Respondent 20). Although accessibility of allied health and complimentary therapies were challenging due to geographical isolation, these components were equally valued.

Support of carers in bereavement represented an ongoing connection with Little Haven long after the death of the patient: ‘If I am really upset. . .I can call and talk to anybody [at Little Haven]’ (Bereaved Carer 3). Respondents to the Carer Satisfaction Survey consistently reported highly positive scores (89–97% affirmative) of Little Haven’s timeliness, inclusion in decision-making, respect for care preferences, clear information, respect for cultural and personal beliefs, responsiveness and support for emotional and spiritual needs. An overall score of ‘extremely satisfied’ was reported by 91% of 116 respondents across a 4-year period. These findings are reported in-depth separately to this paper.

Despite some initial unawareness of Little Haven’s services by some patients and carers, it was generally acknowledged by others that Little Haven is well known and well regarded in the community. Their profile was lifted through public fundraising but there was a sense of frustration at the apparent necessity to allocate significant resources for this:

They have so many volunteers but so much of their energy is taken up fundraising so that they can supply all of this at no charge. It was just amazing . . . all of the visits, and the care, 24-hour care. That all costs money, to have nurses on hand, all of those things. So much of their energy is taken up fundraising where all of that energy should be put towards supporting people and their families. So if they are funded with more and it is a model that works, we know it works, but it needs to be funded so that their energies can be put into looking after the people and their families, not fundraising. . .everybody knows Little Haven in town. . . (Bereaved Carer 2)

### Staff perspectives

There’s a story. . .you know. Phyllis Little started Little Haven from a need in the community. And her group of women were committed to making it work. And now, here we are 40 years later, with an incredible service. It’s a connection to story. It’s a connection to something real, that ignited this service. (Staff Member 3)

This excerpt captures the foundation of Little Haven’s emergence *from* the local community. The clinical team is the interface through which patients and carers experience the therapeutic aspects of their model of care. Staff members were able to reflect on their perspectives as key facilitators of the model. As with patients and carers, staff described the person-centred, holistic characteristics of the Little Haven model:

It’s truly client centred care . . . it’s what each client needs. We might have a bit of a framework for our care, but we also tailor it to each client and each family based on their needs. . . . The focus is always the client’s needs. (Staff Member 3)

Through a multidisciplinary team approach, complex needs were addressed through a coordinated team of nursing, allied health and complementary therapies within Little Haven; liaison with General Practitioners (GPs), the local hospitals and the Primary Health Network comprised the interagency activities.

Little Haven’s commitment to accepting all patients referred, regardless of expected length of life, financial status or social support was a defining feature of the model:

Little Haven . . . [is] holistic, and a very social-based nursing model of care. That it is early entry, that it’s zero discrimination. It doesn’t matter if people live alone or have carers, it doesn’t matter where people live, as long as it’s in a geographical zone, it doesn’t matter if it’s a shanty, to a mansion. And everybody’s treated exactly the same. And that because we don’t have any means testing for that, our services are free to every single person, there’s no limitations to who we can care for. And everybody gets the same care and access to care. (Staff Member 3)

For Little Haven, those living alone are supported to remain home if preferred, with average hospital admissions for end-of-life care of <5 days, and, occasionally, deaths at home.

Early engagement benefited patients and carers and was highly valued by staff: ‘Meeting early means that you can build that rapport. . .you get to know them, and they trust you. And you’re able to go through the whole trajectory with them, which is really special’ (Staff Member 6). This was understood in concrete terms as a characteristic of the Little Haven model that differentiated it from many others:

. . .the fact that you’re with people, it’s such an intimate time in their life, you do develop quick rapport. But I think that the experience for the patient is better if they had a longer time to develop that. There is plenty of research, supporting that early access to palliative care improves quality of life and quantity of life. That’s one of the selling points. We’re going to manage your symptoms; you’re going to have a better quality of life. (Staff Member 1)

Early engagement also results in several patients enlisted with Little Haven at any one time who are stable and require minimal support, something not usually found in Australian palliative care services:

We take patients from the minute they ask for us. Nobody is turned away. Generally, a palliative diagnosis is 90 days, the three months thing, and a lot of people don’t recognise palliative care as being as extensive as we do. Our run sheets do actually have stable people on them, and we are doing a lot of holistic caring, really preparing these people spiritually and emotionally. (Staff Member 10)

The model’s flexible, person-centred approach means that when patients’ needs change, the service can respond promptly and appropriately to people with whom they have built rapport and who trust them. Interestingly, as part of caring for carers, Little Haven will attend a home visit of a stable patient when the carer requires it.

Staff observed increased capacity of patients to remain at home, consistent with known preferences. Components of the model that support this include the practical help, clinical advice, responsiveness and 24-h availability across the region, including isolated, rural properties:

So many people talk about how that’s what got them through. They knew if something happened at two o’clock, they could call. (Staff Member 2)

A highly flexible approach was a hallmark feature of the Little Haven model. Staff identified value in being able to move in their work according to the presenting needs of the patient and carer, rather than adhering to a projected, rigid schedule:

I’ve worked for other community nursing organizations, and they’re very strict with how long we get with each person, whereas I love the Little Haven model, which is you’d have a visit, and if that visit’s 15–20 minutes, or if that visit’s two hours, it’s whatever that family needed that day at that time. (Staff Member 3)

Where other services become involved, Little Haven nurses can provide patient-specific training in aspects of home care and this support was sometimes specifically requested by other services, to promote optimal care outcomes for the patient and carer:

. . .it’s good that organizations come to us and say, ‘Could you just talk our workers through what you’d expect of their care in the home?’, and we’re able to say we would like some consistency with the carer in going into the home, we don’t want a different person going in every day. I think that helps upskilling them as an organization. (Staff Member 1)

This expands the care coordination role Little Haven takes from synchronizing services to capacity building. In this sense, the organization has established a place in the local healthcare sector as providing a preferred model of care.

A notable component of Little Haven’s model of care was its bereavement support. Like bereaved carers’ experiences reported above, staff were clear that this support was an essential part of care. The processes are described in Box 2.

**Box 2.** Bereavement care processes.

The day following the death of a patient, a member of the clinical nursing team will visit the family to provide bereavement support. There is also a pack that is sent out in the mail that has a little booklet that just talks about grief. It lets them know that a social worker will be in contact in the next 2 weeks, and that there are some complimentary therapies available to them as well as part of the bereavement support program. Within the 2 weeks, one of our social workers will reach out over the phone. They will talk to the person and assess where they are at, and what might be of assistance for them in terms of bereavement support. . . .The model tries to address that everybody grieves differently, everyone’s circumstances are different. Not everyone necessarily needs six face-to-face sessions. But, you know, just trying to identify with that person collaboratively about what is going to be useful at this time (Staff Member 2).

Little Haven also sent cards to bereaved families at the anniversary of the death and at Christmas, including information about navigating the first Christmas without the deceased. An annual memorial function is also held.

### Volunteers’ perspectives

Volunteers were described by the clinical team as vital to the success of the Little Haven model. Many of those who become involved with Little Haven had been supported by Little Haven, sometimes from decades ago:

Volunteering is such a big part of our community model as well because. . . a lot of services [have] moved away from volunteering because it’s difficult, then managing volunteers is difficult, but what they bring to us is so important. One is a social support system for some of our bereaved who, come back into the into the model as volunteers. They have their own social support. (Staff Member 1)

This was valuable experience to bring, with a strong sentiment of ‘giving back’. After retirement this volunteer was:

. . .always looking for something to do. I was just sitting around at home and another lady was a volunteer. . . she put my name forward and they had the introduction day, so that’s when I joined, been there for the last five years. (Volunteer 5)

There were mixed views about the extent of community awareness of Little Haven in Gympie and surrounds. Their charity shop (the ‘Marketplace’) was a pivotal interface between Little Haven and the community, but even there, awareness varied enormously:

A lot of people know about Little Haven, but a lot of people don’t. We run the Marketplace . . . and a big surprise is how many people ask you what Little Haven is about and where does the money go? (Volunteer 5)

Other public-facing volunteering for Little Haven was also seen as a valuable way to raise awareness, such as the ‘Happy Wrappers’ who wrap Christmas presents in a local shopping centre, using the opportunity to have informal conversations with the broader community. Nevertheless, where Little Haven was known to community members, it was held in high esteem. They saw it as a service of ‘care and compassion . . . shown to the family members’ (Volunteer 9), where locally raised funds remained in the local community. Participants agreed that it is known for going ‘above and beyond’ what might otherwise be provided. This volunteer, who had been a carer for her husband, experienced this first-hand:

The nurses go, you know, they’re 24/7. When my husband was sick and couldn’t believe that the nurses were 24/7 . . . And ‘anytime, just pick up the phone just ring me’ 24/7. . .who gives you that? Some places don’t even give you that, it’s like, ‘Oh, go somewhere else’. (Volunteer 12)

It was acknowledged that the nature of volunteering is changing and that, with time, fewer volunteers were likely to be available to support the work of Little Haven. Some felt that the situation was manageable for now but not indefinitely: ‘While we’re all going, it’s great. But in five years, there’s probably going to be half of us not volunteering. Yeah, so who’s going to replace us?’ (Volunteer 4) The future was seen as a challenge and raising public awareness as critical to facing it. Opportunities were seen in the Marketplace:

I think down at the Marketplace, we need to put more information out, what’s Little Haven? with our signage: Little Haven Marketplace. That could be anything. Palliative care: have you heard that word there or not? ‘Little Haven Marketplace’, it might give people a better idea. (Volunteer 5)

Raising awareness was also about enabling the Gympie community to understand how it is that they have an organization like Little Haven running at all:

. . . everybody in Gympie can know more about Little Haven, what they do and what they stand for, and why we do what we do in Little Haven . . . like, you know why we get the funds when we sell raffle tickets, because that’s how we run Little Haven. Because like, when someone is sick, they bring out the equipment and all that and look after them. We don’t ask for anything. So how are we going to run if you don’t have all these people behind [it]? (Volunteer 12)

### Stakeholders’ perspectives

Organizational stakeholders were clearly complimentary of the quality of clinical care provided by Little Haven:

The standout thing is. . .they have a good skill set, they are very patient focused, and they will do what it takes to try to deliver the care that the patient wants in the patient’s preferred place of death. (Stakeholder 1)

This was demonstrated most clearly in the responsiveness and flexibility of the model of care:

I don’t think that there is any other service that offers that kind of support. A few years ago, I had a palliative patient who. . .was actually under one of the other organizations in town who were purporting to do palliative care, business hours, Monday to Friday. Four o’clock, four-thirty rolled around, they were uncontactable, and it was a woman in her 50s who was going to die. Most of this stuff happens out of hours, and her family needed support, so thankfully, Little Haven took her on, because the other service just couldn’t offer it, and they eventually admitted that they couldn’t offer that. (Stakeholder 5)

This example speaks to the accountability Little Haven has in a regional town where the population is small and the connections are strong; indeed, rejecting immediate access can have a social cost:

Little Haven doesn’t turn anyone away . . . It is a community organization responsive to our community’s needs. It’s pretty hard to turn down someone when you know that you know the next week . . . like we’re buying our jams and going down to the Little Haven shop, and you know it’s very much ‘everybody knows somebody’. You have to be accountable for your actions. (Stakeholder 5)

Strong views were held of the deep-seated, values-driven motivation of the staff in doing the work of Little Haven. The ‘boutique’ character of the organization enabled its immersion in the local community:

When you get too big. . .I’ve seen that sort of change over the years because it’s got bigger, and then it goes, you know, interstate and it then becomes this big monolith. And it loses that genuineness about it, that innocent kind of just loving kind of organization. And that’s how I see Little Haven. These guys are genuine and give from the heart and nothing is too hard. (Stakeholder 1)

The engagement of the community with Little Haven was seen to rely in some part upon the community’s familiarity with the issues of death and dying, particularly in the context of an ageing population:

. . .we’re such a death-denying society. . .so why would you need to. . .understand why Little Haven, what is it? But also, I do think that all communities are now really struggling with what is a community and trying to engage that community spirit and foster it. There is a lot more individualism now. (Stakeholder 5)

Most participants linked this death denial to a lack of awareness of Little Haven, as a key player in the region in matters of dying, death and grief, where interaction with the service elicits increased acceptance of dying. Lack of readiness for the death of a family member impacted upon the subsequent grieving experienced by the bereaved:

. . .if there’s increased grief, because they haven’t got much exposure prior to this point of people dying and losing a loved one, then it requires much more support to be given by the nurses and the volunteers and social workers to try and help carry that family through that event. Support family members post-death as well. So that puts a huge impost on what the service can deliver when there’s more and more people to care for. (Stakeholder 1)

Other participants in this evaluation strongly linked raising awareness of dying and palliative care to lifting the profile of Little Haven. This intersection between service and community was summed up by this GP:

. . .it’s pretty well-regarded and well-thought of and is protective of it. It’s a service that has risen to the needs of the community that I think the community’s proud of. I think they’re leaders in the community. (Stakeholder 7)

## Discussion

The Little Haven model of care demonstrates common service elements which are central to its structure and processes. The components of the model clearly align with those identified across the palliative care sector in Australia (Table 1); while not all services provide all the components, they were clearly identifiable in this study and are considered below. Together, these elements may provide a reduction in the avoidable transfer of home-dwelling patients to hospital during palliative care, which in turn can result in better patient and carer experiences, and efficiencies in the cost of health care.^16^

### Accessibility and early intervention

Services are accessible as early as required in the patient journey, which is noted above to be linked to improved end-of-life outcomes, including alleviation of symptom burden,^3^ reduced pain^4^ and improved satisfaction with care.^5^

### Specialized staff and holistic care

Specialist clinical staff are competent and compassionate, with a reputation for going ‘above and beyond’ in meeting these needs. It addresses the outcomes of being able to ‘improve pain and symptom control, increase quality of life, improve family bereavement, reduce the need for hospital-based care and provide cost-savings for governments’ (Haydon *et al*.,^38^ p. 625).

### Patient- and family-centred approach

Little Haven provides a person-centred, holistic approach to the care and support of patients and their families and carers. It is flexible in responding to changing needs, reflecting that ‘a flexible service. . .allowed carers to maintain “normal” family life activities’ (Youens and Moorin,^16^ p. 276).

### Free to low-cost

The service is free-of-charge to users, made possible through a combination of government funding and local fundraising. From the beginning, the Gympie community has raised hundreds of thousands of dollars in fundraising, to support the existence and function of ‘its’ palliative care service. Its sense of investment in Little Haven is bolstered by the funds remaining with the town and its surrounds, rather than siphoned off to a distant central administrative location. An economic evaluation was undertaken as part of a wider study and will be reported separately.

### Community involvement

The establishment of the model of Little Haven’s community-based palliative care service began in response to tangible needs identified in the Gympie community. Similarly, the local – rather than centralized – governance of the service is highly regarded by most participants as essential to the model. Little Haven recruits and supports a committed cohort of volunteers, many with first-hand experience of these services. Little Haven urges non-professional team members such as friends, relatives, family members, volunteer community members and spiritual/faith leaders to provide assistance to patients and families in any way they can. This mobilization of informal social networks is central to providing holistic, person-centred care. This refers not only to those who volunteer or are known to patients, but to the wider community, whose capacity to engage with fellow citizens is a critical element of responding to ‘less urgent, practical, emotional and spiritual care’ (Stanley and Daddow,^32^ p. 1345).

### Being in the community

This evaluation identified that Little Haven’s *being in the community* goes beyond service provision or even sentiment. This evaluation observed a symbiotic relationship between the organization and the community it supports. Deeply embedded in the community, Little Haven expresses its responsibility to that community. Many of its practices are developed to enable an unfettered response to identified community need.

This symbiotic relationship was made apparent in a number of ways, including how volunteers were at the interface of service and community. With the involvement of so many volunteers over so many years, Little Haven cannot be separated as a distinct entity from the community in which it has developed, but rather reflects its context. With many volunteers having themselves received carer and bereavement support from Little Haven for family members or friends, they not only bring their lived experience, but engender accountability of the service to the community as their representatives; when Little Haven’s volunteers enter a person’s home, they were viewed as representatives of the local community, *not just the service*. Indeed, volunteers were widely regarded as an indispensable component of the model, deeply valued and treated as equal members of the team.

More broadly – and not surprisingly – end-of-life care is not something that is widely known about until it is needed, therefore, patients and carers alike begin to learn about the service offered by Little Haven from the time of admission. This collective lack of readiness for end-of-life issues becomes evident when confronted with the impending death of a family member, which can have a flow on effect upon the grieving to follow. The community engagement strategies implemented by Little Haven bring both its expertise in response to identified community need, and its familiarity with the issues of dying, caregiving, death and grief to a wider conversation. It is actively engaged – beyond fundraising and volunteering – in its annual ‘Little Haven Week’ and memorial service, gratitude walks, public forums and Dying2Know Day; it has an online presence on its website and Facebook page. However, these are not simply information sharing exercises; they are seen as an ongoing community discussion about its particular needs and strategies to address them.

This strongly reflects the principles and practices of Public Health Palliative Care where partnerships between communities and palliative care services are central to their success. Models of care that intentionally pursue building the capacity of local communities can be key partners in, but not controllers of, matters of dying, caregiving and grieving.^39^ This shared sense of responsibility may be more clearly identifiable in places where there is strong local cohesiveness, including regional centres like Gympie. However, it cannot be assumed that this characteristic is automatically a feature of rural communities and absent in metropolitan ones. While Little Haven’s model demonstrates its place as a ‘citizen’ within the regional community it services, community cohesiveness is observable elsewhere.

### The ‘citizen organization’

The notion of a ‘citizen organization’ has been explored in urban geography^40^ ;however, it is not mentioned in the palliative care literature. In Public Health Palliative Care, citizenship is described in terms of *activated* individuals or collectives, whose participation in issues of dying, death and loss is largely – but not exclusively – contingent upon partnerships with palliative care services who promote community development approaches.^21,22^ This is a valuable arrangement in any community; however, the distinctive characteristic of the citizen organization is its inseparability from the community in which it dwells. It emerges from a community in response to self-identified need; it is governed and enacted (or ‘delivered’) by its own citizens (professional or non-professional alike); by integrating person- and family-centred care into its practice, the citizen organization arranges its support of people by identifying existing capacity within families, neighbourhoods and other social networks. Its operational responsiveness characterizes its prioritizing of local need. The citizen organization is fundamentally democratic in its relationship with other community members and eschews institution-centric, top-down approaches. The ‘gravitational pull’ of institutions described by Russell^41^ is monitored and moderated through its accountability to its fellow citizens for its conduct. It brings its familiarity with the issues of dying, death and grief to community conversations, not as the external expert but as a knowledgeable local citizen; it participates in the growing death literacy of the community as member of it.

These are not naïve aspirations for the provision of palliative care in communities. In fact, the citizen organization can be an expression of the ‘realistic utopia’ recently described in the ‘Lancet Commission on the value of death: bringing death back into life’.^42^ The five principles for death, dying and grief in the future (Table 3) go some way to describing the character of a citizen organization and the community in which it dwells in their support of dying, caring and grieving citizens. Based in Public Health Palliative Care approaches that embed the *Ottawa Charter for Health Promotion*,^22^ a citizen organization addresses social determinants of dying well and grieving well through participation in prevention and harm reduction^42^; this includes mobilizing social support networks, ensuring availability and responsiveness of palliative care and bereavement services and promoting social and workplace recognition of healthy dying and healthy grieving. The relational and spiritual nature of dying underpins holistic approaches to support in the experiences of dying, caregiving and grieving, moving beyond service provision mindsets into embedded enablement of social networks of care. The citizen organization promotes the normalization of public discourse on dying, caregiving and grieving; this not only raises awareness but contributes to increased recognition of the value of death in improving experiences around dying, caregiving and grieving.^42^ This element of community-based palliative care service models can be replicated elsewhere – its responsiveness to local need and the community is the critical enabler for wider implementation.

**Table 3.** Principles of a ‘realistic utopia’ for death, dying and grief.^42^

The social determinants of death, dying and grieving are tackled.
Dying is understood to be a relational and spiritual process rather than simply a physiological event.
Networks of care lead support for people dying, caring and grieving.
Conversations and stories about everyday death, dying and grief become common.
Death is recognized as having value.

### Limitations of the model

The synergies between the service and the community bring a reliance upon the resourcefulness – both fiscal and human – of the community to ensure service sustainability; while this has been sufficient to date, it remains an element of risk as socioeconomic status and community capacity vary over time. Ensuring local governance is retained whilst obtaining government funding is another challenging feature of the model.

## Conclusion

The Little Haven model of care cannot be separated from the community in which it has developed, reflecting its deeply embedded quality. Its structures and processes are both derived from community need and inform community awareness. The efforts put into community engagement are a key part of Little Haven’s place as both a provider of services and a citizen organization in the region. In return for the community’s investment – both social and financial – Little Haven provides members with holistic, immersive, multidisciplined, high standard palliative care, free of financial cost to the patient and their family. Community members are offered the experience of being held deeply by their community, as they move towards the end of their life. This care is extended beyond death, in the form of bereavement support and community remembrance.

Where there are cohesive communities with a strong sense of identity and healthcare providers who not simply provide a service but are a clear ‘citizen’ of the community, the holistic care needs of community members *and* the broader social character of the community address the issues of dying, caregiving, death and grief. It is clear in this evaluation that this characteristic permeates throughout Little Haven’s model and is a significant point of difference at the very foundations of its presence in the Gympie community.

## Supplemental Material

sj-docx-1-pcr-10.1177_26323524241260427 – Supplemental material for Exploring the ‘citizen organization’: an evaluation of a regional Australian community-based palliative care service model Supplemental material, sj-docx-1-pcr-10.1177_26323524241260427 for Exploring the ‘citizen organization’: an evaluation of a regional Australian community-based palliative care service model by John Rosenberg, Trudi Flynn, Katharina Merollini, Josie Linn, Doreen Nabukalu and Cindy Davis in Palliative Care and Social Practice

sj-docx-2-pcr-10.1177_26323524241260427 – Supplemental material for Exploring the ‘citizen organization’: an evaluation of a regional Australian community-based palliative care service model Supplemental material, sj-docx-2-pcr-10.1177_26323524241260427 for Exploring the ‘citizen organization’: an evaluation of a regional Australian community-based palliative care service model by John Rosenberg, Trudi Flynn, Katharina Merollini, Josie Linn, Doreen Nabukalu and Cindy Davis in Palliative Care and Social Practice

## Declarations

**Ethics approval and consent to participate:** Ethical approval was granted by the University of the Sunshine Coast Human Research Ethics Committee (#A221806); all participants provided written consent to participate, and a waiver of consent was granted by the Committee for analysis of retrospective anonymous, secondary survey data. All other data have been deidentified for publication.

**Consent for publication:** All participants in the interviews and focus groups granted consent for the use of their deidentified data in this evaluation including publication.

**Availability of data and materials:** Not applicable.
