# Supplementary material for: Exploring the ‘citizen organization’: an evaluation of a regional Australian community-based palliative care service model
Source: Palliat Care Soc Pract. 2024 Jun 24;18:26323524241260427. doi: 10.1177/26323524241260427 (PMC11265238; doi:10.1177/26323524241260427)
Supplement: sj-docx-1-pcr-10.1177_26323524241260427 – Supplemental material for Exploring the ‘citizen organization’: an evaluation of a regional Australian community-based palliative care service model [file sj-docx-1-pcr-10.1177_26323524241260427.docx]

**SUPPLEMENTARY FILE 1: COREQ CHECKLIST**

| **Domain 1: research team and reflexivity** | | |
| --- | --- | --- |
| **Personal characteristics** | | |
| 1. Interviewers/ facilitators | Which author(s) conducted the interviews or focus group? | JR: stakeholder interviews; volunteer focus group; JL: Patient and carer interviews; TF: Staff focus groups. |
| 2. Credentials | What were the researchers’ credentials? | JR, TF, KM, CM: PhD. JL: MSci(Hons), MCounselling; DN: MPubH. |
| 3. Occupation | What was their occupation at the time of the study? | JR: Senior Lecturer, Nursing; TF: Lecturer, Counselling; KM: Lecturer, Health Economics; JL, DN: Research Assistant; CM: Professor, Social Work. |
| 4. Gender | Was the researcher male or female? | JR: Male. TF, KM, JL, DN, CM: Female |
| 5. Experience and training | What experience or training did the researcher have? | JR, TF, CM: PhD-qualified researchers with training and longstanding experience in qualitative and mixed-method research methodologies. KM: PhD-qualified researcher with training and experience in a range of research methodologies, in particular quantitative methods and economic evaluation. JL: Masters-prepared counselling practitioner. DN: Masters-prepared public health practitioner and PhD student under supervision from KM. |
| **Relationship with participants** | | |
| 6. Relationship established | Was a relationship established prior to study commencement? | JR was known to the service prior to commissioning of evaluation through longstanding presence in palliative care sector.  JL was known to the service as a previous employee as counsellor.  TF, KM, DN, CM had no prior relationship. |
| 7. Participant knowledge of the interviewer | What did the participants know about the researchers? | Participants knew where the researchers worked and the purpose of the research as an evaluation of the service model of care. |
| 8. Interviewer characteristics | What characteristics were reported about the interviewer/facilitator? | JR is well-known in the palliative care sector as a palliative care nurse and researcher, and was known professionally by the service Manager. JL was known by some of the staff and volunteers from her previous counselling role. All other researchers were not known to participants. |
| **Domain 2: study design** | | |
| **Theoretical framework** | | |
| 9. Methodological orientation and theory | What methodological orientation was stated to underpin the study? | The study was atheoretical and underpinned by a pragmatic approach enabling research design to be applied to service evaluation. |
| **Participant selection** | | |
| 10. Sampling | How were participants selected? (e.g. purposive, convenience, consecutive, snowball) | Staff; volunteers; stakeholders: purposive sample.  Current patients and carers: purposive sample.  Bereaved carers: convenience sample of retrospective data. |
| 11. Method of approach | How were participants approached? (e.g. face to face, telephone, mail, e-mail) | Staff; volunteers; stakeholders: email; hard copy notices in staff/volunteer areas in LH offices.  Current patients and carers: emailed via LH administration, notices on notice board of LH offices.  Bereaved carers: n/a |
| 12. Sample size | How many participants were in the study? | Staff n=10; volunteers n=13; stakeholders n=8.  Current patients n=6; carers n=3; bereaved carers (interviews) n=7; bereaved carers (retrospective survey) n=116. |
| 13. Non-participation | How many people refused to participate or dropped out? Reasons? | One patient refusal (following consent) due to rapid deterioration in health status. |
| **Setting** | | |
| 14. Setting of data collection | Where was the data collected? (e.g. home, clinic, workplace) | Focus groups for staff and volunteers were conducted separately, away from LH offices in meeting rooms.  Stakeholders were interviewed using Zoom™.  Current patients and carers, and bereaved carers were interviewed in their homes (at their request). |
| 15. Presence of non-participants | Was anyone else present besides the participants and researchers? | No. |
| 16. Description of sample | What are the important characteristics of the sample? (e.g. demographic data, date) | Brief demographic data were collected about selected participant groups. |
| **Data collection** | | |
| 17. Interview guide | Were questions, prompts, guides provided by the authors? Was it pilot tested? | Guidance questions were developed and used during the interviews and focus groups. These were carefully reviewed within the research team prior to use. |
| 18. Repeat interviews | Were repeat interviews carried out? If yes, how many? | No repeat focus group or interviews were required. |
| 19. Audio/visual recording | Did the research use audio or visual recording to collect the data? | Interviews and focus group sessions were audio-recorded or recorded when conducted via Zoom™. |
| 20. Field notes | Were field notes made during and/or after the interview or focus group? | Yes. |
| 21. Duration | What was the duration of the interviews or focus group? | Focus group duration ranged from 60 to 75 minutes and interviews ranged in duration from 22 to 82 minutes. |
| 22. Data saturation | Was data saturation discussed? | Data saturation was not discussed. |
| 23. Transcripts returned | Were transcripts returned to participants for comment and/or correction? | Transcripts were not returned to participants. |
| **Domain 3: analysis and findings** | | |
| **Data analysis** | | |
| 24. Number of data coders | How many data coders coded the data? | Two researchers independently coded the data from each source. |
| 25. Description of the coding tree | Did authors provide a description of the coding tree? | No. |
| 26. Derivation of themes | Were themes identified in advance or derived from the data? | The study utilised areas of evaluation focus (after Blumer’s sensitizing concepts) however themes were derived from the data. |
| 27. Software | What software, if applicable, was used to manage the data? | n/a |
| 28. Participant checking | Did participants provide feedback on the findings? | Member checking is contested and was not utilised in this study. |
| **Reporting** | | |
| 29. Quotations presented | Were participant quotations presented to illustrate the themes/findings? Was each quotation identified? (e.g. participant number) | Yes. |
| 30. Data and findings consistent | Was there consistency between the data presented and the findings? | We endeavoured to report the study findings in a clear, consistent manner in order to accurately reflect the data that have been collected. |
| 31. Clarity of major themes | Were major themes clearly presented in the findings? | The Findings section reports themes from each participant group. The major themes are reported in the Discussion. |
| 32. Clarity of minor themes | Is there a description of diverse cases or discussion of minor themes? | Minor themes not explored in this manuscript due to word count restriction. |
