# Supplementary material for: Exploring the ‘citizen organization’: an evaluation of a regional Australian community-based palliative care service model
Source: Palliat Care Soc Pract. 2024 Jun 24;18:26323524241260427. doi: 10.1177/26323524241260427 (PMC11265238; doi:10.1177/26323524241260427)
Supplement: sj-docx-2-pcr-10.1177_26323524241260427 – Supplemental material for Exploring the ‘citizen organization’: an evaluation of a regional Australian community-based palliative care service model [file sj-docx-2-pcr-10.1177_26323524241260427.docx]

**SUPPLEMENTARY FILE 2: LITTLE HAVEN CARER SATISFACTION SURVEY TOOL**

1. **Who referred you to Little Haven?**

⬜ Self ⬜ Doctor ⬜ Hospital ⬜ Other (please specify): _____________________

1. **Do you feel that the involvement of Little Haven was undertaken in a timely manner?**

⬜ Yes ⬜ No

If NO, please explain your reasons: _________________________________________________

1. **Did the staff of Little Haven include both you and your loved one in the decision-making process regarding their care?**

⬜ Yes ⬜ No

1. **Did our staff respect the preferences expressed by the patient and their family during the time of our care?**

⬜ Yes ⬜ No

1. **Were you given clear, comprehensive information aby our staff with regard to the care of your loved one in a way that you could understand?**

⬜ Yes ⬜ No

1. **Were your cultural and personal beliefs recognised and respected by our service?**

⬜ Yes ⬜ No

1. **How satisfied were you with the level of care provided by the Little Haven staff?**

⬜ Extremely satisfied ⬜ Satisfied ⬜ Not satisfied

1. **If there are any aspects of our service that you feel needs improvement, please give details below:** ______________________________________________________________________________
2. **How well were your spiritual/emotional needs supported by Little Haven?**

1 2 3 4 5

Please circle

(1 being – Not supported & 5 being – Greatly supported)

1. **Were your telephone contacts with Little Haven staff answered promptly and effectively?**

⬜ Yes ⬜ No

If NO, please comment: __________________________________________________________

1. **Were you made aware of all aspects of our service including:**
   - Nursing staff on call 24 hours a day? ⬜ Yes ⬜ No
   - Free loan of equipment? ⬜ Yes ⬜ No
   - Bereavement support? ⬜ Yes ⬜ No
   - Complementary therapies? (eg: Massage, Reiki, Bowen Therapy) ⬜ Yes ⬜ No
   - Respite Volunteers ⬜ Yes ⬜ No
2. **If you used any of our Little Haven services, could you please tick the ones that you used:**
   - 24 hour on call service ⬜
   - Equipment loan ⬜
   - Bereavement support ⬜
   - Complementary therapies? ⬜
   - Respite Volunteers ⬜
3. **Thinking about our complementary therapies, how useful did you find the ones you used?**

1 2 3 4 5

Please circle

(1 being – Not useful & 5 being – Very useful)

1. **Think about our respite volunteers, how beneficial did you find them?**

1 2 3 4 5

Please circle

(1 being – Not beneficial & 5 being – Very beneficial)

**Additional comments:** ______________________________________________________________________________________________________________________________________________________________________________________________________________________________________________________________________________

Thank you for your time.
